# Use of intravitreal fluocinolone acetonide implant in inflammatory macular oedema

**DOI:** 10.1038/s41433-026-04381-9

**Published:** 2026-03-19

**Authors:** Chloé Gullon, Sandra Elbany, Nicolas Romain-Scelle, Carole Burillon, Corinne Dot, Thibaud Mathis, Yasmine Serrar, Mariam Ghazaryan, François Devin, Laurent Kodjikian

**Affiliations:** 1 Service d’Ophtalmologie, Hôpital universitaire de la Croix-Rousse, Hospices Civils de Lyon, Lyon, France; 2 Service d’Ophtalmologie, Hôpital universitaire Edouard Herriot, Hospices Civils de Lyon, Lyon, France; 3 Service de Biostatistiques, Hôpital universitaire de Lyon Sud, Hospices Civils de Lyon, Lyon, France; 4 Université Claude Bernard Lyon 1, Lyon, France; 5 Académie de santé militaire du Val de Grâce, Paris, France; 6 Laboratoire UMR-CNRS 5510 MATEIS, INSA, Université Claude Bernard Lyon 1, Villeurbanne, France; 7 Teona Ophthalmology Clinic, Yerevan, Armenia; 8 Centre Monticelli-Paradis, Clinique Juge, Marseille, France

**Keywords:** Retinal diseases, Diseases, Eye diseases

## Abstract

**Background/objectives:**

To assess the effectiveness and safety of 0.19-mg fluocinolone acetonide implant (FAc-implant) in inflammatory macular oedema (MO).

**Subjects/methods:**

This observational, retrospective, multicentre cohort included consecutive eyes treated with FAc-implant between June 2020 and January 2024 for recurrent uveitic or postoperative MO requiring at least two prior dexamethasone-implants (DEX-implants) and ≥6 months of follow-up. The primary endpoints were best-corrected visual acuity (BCVA) and central macular thickness (CMT) at 6, 12, 18, and 24 months. The secondary endpoints were anatomical and functional outcomes, additional treatments and safety outcomes (intraocular pressure (IOP), ocular hypertension (OHT)).

**Results:**

A total of 73 eyes were included, with a mean follow-up of 16.8 (8.4) months. The median difference in BCVA was +4.0 [0.0;10.0] (*p* < 0.001), +2.0 [0.0;9.0] (*p* = 0.006), +5.0 [0.0;10.0] (*p* = 0.010) and +5.0 [0.0;9.0] (*p* = 0.043) letters at 6, 12, 18 and 24 months. The median difference in CMT was -71.0 [-181.3;-25.5] (*p* < 0.001), -71.0 [-195.0;-30.5] (*p* < 0.001), -75.0 [194.0;-43.0] (*p* < 0.001) and -154.0 [-253.0;-110.0] (*p* < 0.001) μm at 6, 12, 18 and 24 months. Over the follow-up, 38 eyes (52.1%) did not require any additional intravitreal treatment. At 12 months after FAc-implant, the probability of not receiving a rescue injection of DEX-implant was 54.0% [43.0%;67.0%]. Eight eyes (11.0%) developed OHT (IOP ≥ 25 mmHg or an increase in IOP ≥ 10 mmHg), mostly resolved by IOP-lowering eye drops; two eyes required surgery.

**Conclusions:**

This real-life study shows the functional and anatomical effectiveness of the FAcimplant in recurrent inflammatory MO, with limited adverse effects and a reduced treatment burden.

## Introduction

Inflammatory macular oedema (MO), including uveitic and postoperative MO, is one of the most important causes of reduced visual acuity. Given the frequency and the blinding potential of these situations, the challenge is to provide rapid, appropriate, and sustainable treatment.

It is estimated that a third of uveitis patients will develop MO, and that 40% of vision loss in uveitis is due to MO [1]. Corticosteroids, administered either locally (topically, intravitreally, or periocularly) or systemically, remain the cornerstone of treatment for uveitic MO, sometimes supplemented by the use of immunosuppressants. Postoperative MO is generally described as occurring in the first few months following ophthalmic surgery, with a frequency of 0.2 to 2% for cases complicating cataract surgery [2]. Although there is no consensus regarding the management of postoperative MO, a therapeutic escalation approach is generally used. The first line of treatment consists in local non-steroidal anti-inflammatory drugs, sometimes combined with topical corticosteroids and acetazolamide. If this fails, corticosteroids are injected periocularly or intravitreally. Despite a difference in the cause of MO, some shared pathophysiological mechanisms between uveitic and postoperative MO have been reported: both arise from the rupture of the internal or external blood-retinal barrier, involving cytotoxic and vasogenic phenomena as well as common pro-inflammatory mediators [3, 4]. However, phototoxicity and a traction involvement of the vitreous have also been described specifically in postoperative MO [5, 6]. These shared mechanisms may in part explain the use of intravitreal injection of corticosteroids for both causes of MO, the most commonly used being the dexamethasone implant (DEX-implant). However, given the pharmacokinetics of the molecule (extended-release implant for a period of up to 6 months), recurrences often occur after 4-6 months. Fluocinolone acetonide implant 0.19-mg (FAc-implant; Iluvien^®^, Alimera Sciences Ltd, Aldershot, UK), delivering 0.2 µg/day into the vitreous body and releasing very small quantities of the active substance continuously over a long period of time, is a steroid that has recently been approved for the prevention of relapse in recurrent non-infectious uveitis. It has shown favourable results in randomised controlled trials [7, 8], reporting an improvement in visual acuity for up to 3 years in uveitic MO and a decrease in treatment burden. As this molecule has recently been approved for uveitic indications (not yet regulated for postoperative indications), current practice is moving towards an increased use in these inflammatory conditions [9]. However, data on the effectiveness of the FAc-implant in both uveitic and postoperative MO remain scarce.

The primary objective of the present study was to evaluate the functional (best-corrected visual acuity, BCVA) and anatomical (central macular thickness, CMT) effectiveness of the FAcimplant for inflammatory MO (uveitic and postoperative) in real-life conditions at 6, 12, 18, and 24 months. The secondary objectives were to describe functional responders, analyse the effect of the FAc-implant on intra-eye fluctuations in BCVA and CMT, and report follow-up and safety data such as the need for additional treatment.

## Materials/ subjects and methods

### Study design and population

This retrospective cohort study was conducted in four tertiary reference centres in Lyon, France

(*Hôpital Croix-Rousse, Hôpital Edouard-Herriot, Hôpital Lyon-Sud, Hôpital Spécialisé des Armées Desgenettes*) to evaluate the functional and anatomical effectiveness of the FAc-implant for inflammatory MO in real-life conditions. All consecutive patients who received a FAcimplant for inflammatory MO between June 2020 and January 2024, with a minimum of 6 months follow-up after FAc-implant, were included (Supplementary Fig. 1). Consecutive inclusion was performed by searching for the keyword ‘Iluvien’ on the healthcare software that is common to all centres; each file was accessed by chronological order of intervention and manually reviewed. The inclusion criteria were: age ≥18 years, recurrent inflammatory MO (non-infectious uveitic or postoperative) requiring at least two DEX-implants prior to FAcimplant, and a follow-up of at least 6 months after FAc-implant. Patients receiving bilateral FAc-implants were also eligible for inclusion. The exclusion criteria were: MO due to another cause (diabetic, central retinal vein occlusion, etc.), severe glaucoma or uncontrolled ocular hypertension (OHT) needing >3 antiglaucoma drops, and poor response to prior DEX-implants, defined as a reduction of less than 20% in CMT. The use of systemic corticosteroids, and/or immunosuppressants, and/or biologics was not an exclusion criterion. All eyes had documented good response to at least two DEX-implants prior to FAc-implant. The study was conducted in accordance with the Declaration of Helsinki and was approved by the ethics committee of the *Hospices civils de Lyon* (IRB 00013204). All included patients received written information and gave their consent.

### Treatment initiation and follow-up

According to the most recent international guidelines, FAc-implant treatment could be introduced shortly after the DEX-implant, generally 0 to 4 weeks after the last injection [10, 11]. The intravitreal implantation was carried out under aseptic conditions using the standard preloaded sterile single-use FAc-implant 0.19-mg applicator with a 25-gauge needle administered through the pars plana within the inferotemporal quadrant and 4 mm from the limbus. FAc-implant was performed after the patient was clearly informed. The patients were then followed-up at 1 month, 3 months, and then every 3 months after FAc-implant. In case of additional FAc-implant during the follow-up, only the data concerning the first FAc-implant were considered for analysis.

### Data collection

The patients’ demographic (age, sex) and MO-related data (type, aetiology, duration, previous treatments…) at baseline (i.e. prior to FAc-implant) were collected from the medical records.

The following ophthalmological data were also collected at baseline and at each follow-up visit: BCVA measured using the early treatment diabetic retinopathy study (ETDRS) scale, intraocular pressure (IOP), and spectral domain-optical coherence tomography (SD-OCT) to calculate CMT and retinal nerve fibre layer (RNFL). BCVA, CMT, IOP, and RNFL were expressed as values at each follow-up visit or as changes from baseline. The ophthalmological data at 1 month corresponded to the last values recorded at 1 month ± 15 days. For the 3-month follow-up, BCVA, CMT, IOP, and RNFL values retained were the last values recorded between 1.5 months and 4.5 months. The same rule (±1.5 months) was applied for subsequent followup visits (every 3 months). Finally, the need for additional treatment was also collected at each follow-up visit. The prescription of additional DEX-implants, anti-vascular endothelial growth factors (anti-VEGF; ranibizumab, faricimab, or aflibercept), or corticosteroid eye drops was decided at the discretion of the ophthalmologist and was generally administered in case of recurrent MO on SD-OCT, and/or significantly reduced visual acuity, and/or recurrence of uveitis.

### Primary and secondary outcomes

The primary outcome was the change in BCVA in letters, using the EDTRS scale, and the change in CMT in micrometres (µm) between baseline (i.e. prior to FAc-implant) and the follow-up visits at 6, 12, 18, and 24 months. Several secondary outcomes were then considered. In terms of functional and anatomical outcomes, the proportion of eyes showing a functional response (i.e. defined as a BCVA gain ≥5 letters) at any timepoint during follow-up was evaluated. Then, the intra-eye fluctuations in BCVA and CMT, overall and for each MO group (uveitic and postoperative), were analysed one year before and one year after the FAc-implant.

In terms of follow-up and safety, the need for additional treatment was described and the occurrence of any adverse event was reported. For IOP results, OHT was defined as IOP ≥ 25 mmHg or an increase in IOP ≥ 10 mmHg after FAc-implant.

### Statistical analyses

Variable descriptions were conducted using means (standard deviation (SD)) or medians (interquartile range, [IQR]) for continuous variables when appropriate and number (%) for categorical variables, at baseline and for each follow-up visit timepoint. For the primary outcome, changes in BCVA and CMT between each prespecified timepoint and baseline were tested using the Wilcoxon signed rank test for paired data, with p-values adjusted following the Holm’s method to control for the family-wise error rate. For the secondary outcomes, a generalised additive model for location, scale, and shape (GAMLSS) was used to assess the change in intra-eye fluctuations for each outcome (BCVA and CMT) before and after FAcimplant. This model incorporated both a regression for the mean and for the SD of the outcome. The regression for the SD included a random intercept per eye to account for repeated measurements and the FAc-implant (binary) as fixed effect. The change in intra-eye fluctuations was estimated using the coefficient (with its 95% confidence interval [95%CI]) associated with the FAc-implant variable. Using the regression for the mean outcome of the same model, the effect of confounding factors (aetiology, other associated cause of MO, and tractional epiretinal membrane) on the effect of FAc-implant was tested. The analysis was restricted up to one year after the FAc-implant. The modelling was done on the total cohort. Then, in order to describe the time spent without rescue therapy after FAc-implant, the Kaplan-Meier method was used to estimate survival until the first DEX-implant. Finally, the association between increased IOP (defined as IOP ≥21 mmHg) before and after FAc-implant was tested using McNemar’s test. All analyses were conducted in R 4.4.0 with the packages gtsummary and GAMLSS. The alpha risk was set to 0.05 unless otherwise stated.

## Results

### Patients’ characteristics at baseline

A total of 73 eyes (42.5% uveitic MO, 57.5% postoperative MO) from 62 patients were included. Patients’ characteristics at baseline, prior to FAc-implant, are reported in Table 1. The mean MO duration before FAc-implant was 50.9 (59.7) months: 75.7 (92.8) months in the uveitic group and 38.5 (26.8) months in the postoperative group. Confounding factors included the presence of a tractional retinal membrane in 13 eyes (17.8%) and the presence of another associated cause of MO in 7 eyes (9.6%). MO in the uveitic group was idiopathic in 14 (46.7%) eyes. Thirteen eyes (41.9%) from the uveitic group were under systemic treatment before FAcimplant: 4 (30.8%) with corticosteroids, 3 (23.1%) with immunosuppressants, and 6 (46.2%) with a combination of both. In the postoperative group, 11 (26.8%) MO followed retinal detachment. At baseline, in the total cohort, median BCVA was 69.5 [60.0;78.0] letters, median CMT was 409.0 [341.0;488.0] μm, median IOP was 11.0 [9.0;13.0] mmHg, and median RNFL was 104.0 [90.0;120.0] μm. The mean follow-up duration was 16.8 (8.4) months. Overall, 7 eyes (9.6%) were lost to follow-up (4 failed to show up for follow-up visits and 3 were followed-up by their treating ophthalmologist but their data could not be retrieved; Supplementary Fig. 1).

**Table 1:** Patients’ demographics and macular oedema-related data before FAc-implant in the total cohort and according to macular oedema type.

	Total cohort (*n* = 73)	Uveitic MO (*n* = 31)	Postoperative MO (*n* = 42)
**Sex, female, n (%)**	32 (43.8)	16 (21.9)	16 (21.9)
**Age at FAc-implant, years, mean (SD)**	68.1 (12.4)	66.1 (16.5)	69.7 (8.2)
**MO duration before FAc-implant, months, mean (SD)**	50.9 (59.7)	75.7 (92.8)	38.5 (26.8)
**Presence of confounding factor, n (%)**			
Tractional epiretinal membrane	13 (17.8)	7 (9.6)	6 (8.2)
Other associated cause of MO	7 (9.6)	6 (8.2)	1 (1.4)
**Uveitis type, n (%)**			
Unknown		1	
Anterior		28 (93.3)	
Intermediate		25 (83.3)	
Posterior		21 (67.7)	
Panuveitis		19 (63.3)	
**Uveitis aetiology, n (%)**			
Unknown		1	
Idiopathic		14 (46.7)	
Sarcoidosis		5 (16.7)	
Vogt-Koyanagi-Harada disease		2 (6.7)	
Post-infection (tuberculosis, VZV)		5 (16.7)	
HLA-B27+		1 (3.3)	
Multifocal choroiditis		2 (6.7)	
Sympathetic ophthalmia		1 (3.3)	
Reiter’s syndrome		1 (3.3)	
**Inflammatory signs, n (%)**			
Unknown		1	
Vasculitis		20 (66.7)	
Optic nerve swelling		20 (66.7)	
**History of systemic treatments, n (%)**			
No		18 (58.1)	
Yes		13 (41.9)	
Corticosteroids only		4 (30.8)	
IS treatment only		3 (23.1)	
Corticosteroids + IS		6 (46.2)	
**Number of recurrences, one year before FAc-implant, mean (SD)**		1.3 (1.8)	
**Surgery type, n (%)**			
Unknown			1
Cataract			10 (24.4)
Retinal detachment			11 (26.8)
Epiretinal membrane			3 (7.3)
Macular hole			2 (4.9)
Filtering surgery			1 (2.4)
Cataract + retinal detachment			3 (7.3)
Cataract + epiretinal membrane			10 (24.4)
Retinal detachment + epiretinal membrane			1 (2.4)
**Pseudophakic eyes before FAc-implant**	73 (100.0)	31 (42.5)	42 (57.5)
**IOP-related data following DEX-implant, before FAc implant, n (%)**			
Glaucoma			
Unknown	4	0	4
Yes	14 (20.3)	5 (7.3)	9 (13.0)
No	55 (79.7)	26 (37.7)	29 (42.0)
Number of IOP-lowering drops			
0	44 (60.3)	24 (32.9)	20 (27.4)
1	12 (16.4)	0	12 (16.4)
2	13 (17.8)	6 (8.2)	7 (9.6)
3	4 (5.5)	1 (1.4)	3 (4.1)
History of IOP-lowering surgery	4 (5.5)	3 (4.1)	1 (1.4)
History of SLT	8 (11.0)	1 (1.4)	7 (9.6)
History of IOP ≥ 21 mmHg	22 (30.1)	8 (11.0)	14 (19.2)
History of OHT (IOP ≥ 25 mmHg or increase in IOP ≥ 10 mmHg)	12 (16.4)	4 (5.5)	8 (11.0)
**Treatments received before FAc-implant**			
Sub-conjunctival corticosteroid or intravitreal antiVEGF, n (%)			
Betamethasone	18 (24.7)	11 (15.1)	7 (9.6)
Triamcinolone	24 (32.9)	15 (20.6)	9 (12.3)
Anti-VEGF (ranibizumab or aflibercept)	9 (12.3)	6 (8.2)	3 (4.1)
Number of DEX-implants, n (%)			
Unknown, but > 2	1	1	0
2 –5	28 (38.9)	9 (12.5)	19 (26.4)
5 – 10	24 (33.3)	9 (12.5)	15 (20.8)
10 – 20	19 (26.4)	12 (16.7)	7 (9.7)
> 20	1 (1.4)	0	1 (1.4)
Number of DEX-implants, mean (SD)			
Since the onset of the disease	7.8 (4.8)	9.2 (5.5)	6.7 (4.0)
One year before FAc-implant	3.0 (0.8)	2.9 (0.7)	3.1 (0.8)
DEX-implant treatment duration, months, n (%)			
Unknown, but > 6	3	3	0
[6–12]	18 (25.7)	7 (10.0)	11 (15.7)
[12–24]	23 (32.9)	7 (10.0)	16 (22.9)
[24–36]	9 (12.9)	2 (2.9)	7 (10.0)
> 36	20 (28.6)	12 (17.1)	8 (11.4)
Interval time between the last two DEX-implants, months, mean (SD)	3.6 (0.8)	3.6 (0.8)	3.6 (0.8)
Interval time between last DEX-implant and FAc-implant, months, mean (SD)	3.7 (1.3)	3.5 (1.5)	3.9 (1.2)
**Outcome measures at baseline**			
BCVA, letters, mean (SD)	65.3 (15.3)	65.3 (15.5)	65.3 (15.3)
BCVA, letters, median [IQR]	69.5 [60.0;78.0]	67.0 [60.0;78.0]	70.0 [55.0;78.0]
CMT, µm, mean (SD)	425.4 (110.7)	375.5 (80.6)	462.3 (116.2)
CMT, µm, median [IQR]	409.0 [341.0;488.0]	383.0 [321.0;417.0]	468.0 [365.0;534.0]
IOP, mmHg, mean (SD)	11.4 (4.2)	10.8 (4.6)	11.9 (3.8)
IOP, mmHg, median [IQR]	11.0 [9.0;13.0]	10.0 [8.0;12.0]	11.0 [10.0;13.0]
RNFL, µm, mean (SD)	104.2 (24.7)	110.0 (28.4)	99.2 (20.2)
RNFL, µm, median [IQR]	104.0 [90.0;120.0]	104.0 [91.5;126.5]	104.0 [88.8;110.3]

*%* percentage, *anti-VEGF* anti-vascular endothelial growth factor, *BCVA* best-corrected visual acuity, *CMT* central macular thickness, *DEX-implant* dexamethasone implant, *FAc-implant* fluocinolone acetonide implant, *HLA* human leukocyte antigen, *IOP* intraocular pressure, *IQR* interquartile range, *IS* immunosuppressants, *MO* macular oedema, *n*number, *OHT* ocular hypertension, *RNFL* retinal nerve fibre layer, *SD* standard deviation, *SLT* selective laser trabeculoplasty, *VZV* varicella zoster virus.

### Anatomical and functional primary outcomes

The median BCVA significantly improved and the median CMT significantly decreased at every 3-month follow-up visit compared to baseline (Supplementary Table 1). The median difference in BCVA was +4.0 [0.0;10.0] letters at 6 months (*p* < 0.001), +2.0 [0.0;9.0] letters at 12 months (*p* = 0.006), +5.0 [0.0;10.0] letters at 18 months (*p* = 0.010), and +5.0 [0.0;9.0] letters at 24 months (*p* = 0.043). The median difference in CMT was -71.0 [-181.3;-25.5] μm at 6 months (*p* < 0.001), -71.0 [-195.0;-30.5] μm at 12 months (*p* < 0.001), -75.0 [-194.0;-43.0] μm at 18 months (*p* < 0.001), and -154.0 [-253.0;-110.0] μm at 24 months (*p* < 0.001; Table 2).

**Table 2:** Best-corrected visual acuity and central macular thickness at baseline, 6 months, 12 months, 18 months, and 24 months of follow-up in the total cohort.

	M0 *N* = 73	M6 *N* = 71	M12 *N* = 53	M18 *N* = 33	M24 *N* = 13
**Mean BCVA, letters (SD)**	65.3 (15.3)	69.6 (15.0)	70.5 (14.9)	72.5 (10.3)	73.5 (8.4)
**Median BCVA, letters [IQR]**	69.5 [60.0;78.0]	75.0 [62.0;80.0]	75.0 [65.0;80.0]	75.0 [68.0;79.0]	75.0 [67.0;79.0]
**Median difference in BCVA, letters [IQR]**	0 [0.0;0.0]	4.0 [0.0;10.0] **p** < **0.001**	2.0 [0.0;9.0] **p** = **0.006**	5.0 [0.0;10.0] **p** = **0.010**	5.0 [0.0;9.0] **p** = **0.043**
**Mean CMT**, µ**m (SD)**	425.4 (110.7)	317.7 (74.8)	312.4 (77.5)	308.2 (61.8)	286.2 (42.7)
**Median CMT**, µ**m [IQR]**	409.0 [341.0;488.0]	305.0 [271.0;341.0]	302.0 [268.0;342.0]	302.0 [268.0;344.0]	283.0 [259.0;300.0]
**Median difference in CMT**, µ**m [IQR]**	0 [0.0;0.0]	-71.0 [-181.3;-25.5] **p** < **0.001**	-71.0 [-195.0;-30.5] **p** < **0.001**	-75.0 [-194.0;-43.0] **p** < **0.001**	- 154.0 [-253.0;-110.0] **p** < **0.001**

*P*-values were calculated using Wilcoxon signed rank test and corrected following Holm’s method to control for the family-wise error rate.

*BCVA* best-corrected visual acuity, *CMT* central macular thickness, *IQR* interquartile range, *M* month of follow-up visit, *N* number of eyes analysed, *SD* standard deviation.

### Anatomical and functional secondary outcomes

Overall, 53 eyes (72.6%) were functional responders (BCVA gain ≥ 5 letters), 27 eyes (37.0%) had a ≥ 10-letter improvement, and 15 eyes (20.5%) had a ≥ 15-letter improvement at one or more timepoints during follow-up.

**Fig. 1:**
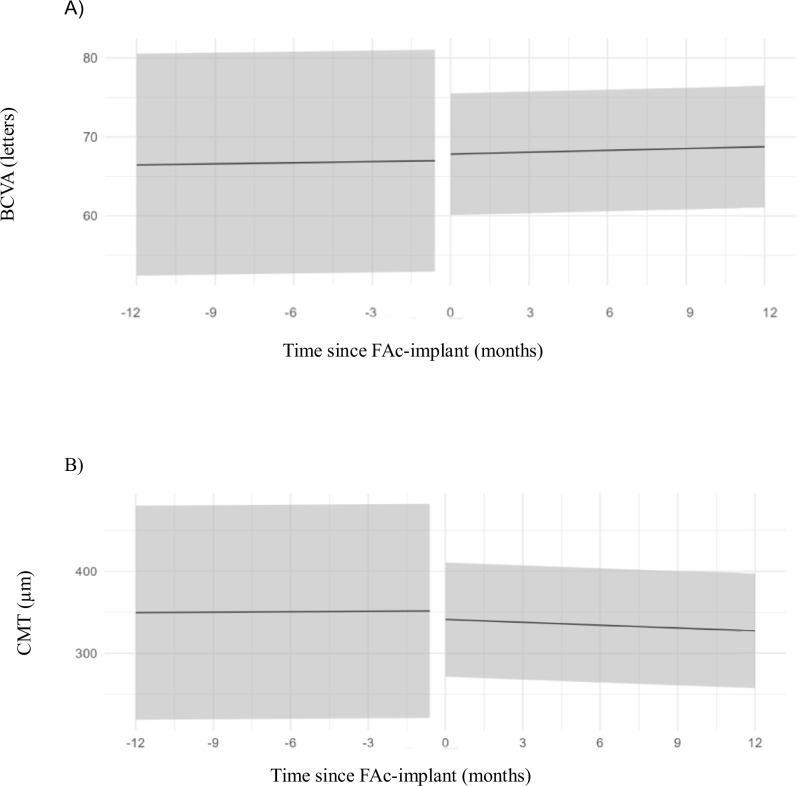
Fluctuations in best-corrected visual acuity and central macular thickness one year after FAc-implant compared to one year before. The solid lines represent the mean and the shaded interval around the solid lines model the intra-eye fluctuations (SD) in BCVA (**A**) and CMT (**B**) for the total cohort. A generalised additive model for location, scale, and shape (GAMLSS) was used to assess the change in intra-eye fluctuations for each outcome (BCVA and CMT) before and after FAcimplant. This model incorporated both a regression for the mean and for the SD of the outcome. The regression for the SD included a random intercept per eye to account for repeated measurements and the FAc-implant (binary) as fixed effect. The change in intra-eye fluctuations was estimated using the coefficient (with its 95% confidence interval [95%CI]) associated with the FAc-implant. The pooled effect of FAc-implant on intra-eye fluctuation (SD) in BCVA was -0.6 [-0.7;-0.5] (*p* < 0.001). The pooled effect of FAc-implant on intra-eye fluctuation (SD) in CMT was -0.6 [-0.7;-0.5] (*p* < 0.001) (Supplementary Table 2). BCVA= best-corrected visual acuity, CI confidence interval, CMT central macular thickness, FAc-implant fluocinolone acetonide implant, GAMLSS generalised additive model for location, scale and shape, SD standard deviation.

In the total cohort, a reduction in intra-eye fluctuations (represented by the change in SD) for both BCVA (SD change: -0.6 [-0.7;-0.5], *p* < 0.001) and CMT (SD change: -0.6 [-0.7;-0.5], *p* < 0.001) was found after FAc-implant compared to before (Fig. 1). The aetiology (uveitis or postoperative) did not have a significant effect on the change in mean BCVA or CMT after FAcimplant (*p* = 0.3 and *p* = 0.2, respectively), nor did the presence of another associated cause of MO (*p* = 0.6 and *p* = 0.5, respectively) or the presence of a tractional epiretinal membrane (*p* = 0.6 and *p* = 0.9; Supplementary Table 2).

### Follow-up and safety secondary outcomes

In terms of additional treatments, 2 eyes received periocular corticosteroid injections and 3 eyes received intravitreal anti-VEGF (2 as adjunctive treatment to DEX-implant) during follow-up. Ten eyes received topical dexamethasone, mainly to treat recurrent anterior uveitis. No topical non-steroidal treatment was administered during the follow-up period. Among the 13 uveitic eyes that received systemic treatment before the FAc-implant (Table 1), treatment was reduced or discontinued in 7 (53.8%) of them. For 2 eyes, treatment was not modified while a change in immunosuppressant was observed in 2 eyes. Therapeutic escalation through the introduction of an immunosuppressant occurred in 2 eyes. Among the 10 eyes that received oral corticosteroid treatment (alone or in combination with immunosuppressants) prior to FAcimplant (Table 1), the average dose decreased from 6.4 mg to 3.8 mg at the last follow-up visit.

**Fig. 2:**
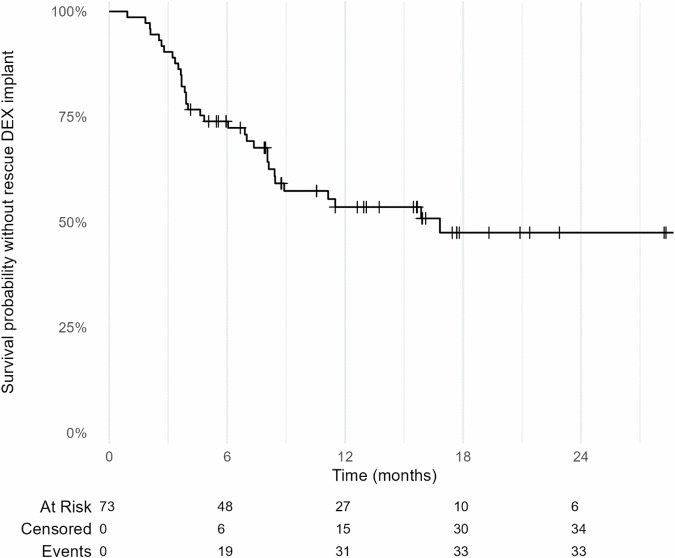
Survival without DEX-implant following FAc-implant according to Kaplan-Meier curve. DEX-implant dexamethasone implant, FAc-implant fluocinolone acetonide implant.

Overall, during the follow-up period, 38 eyes (52.1%) did not require any additional intravitreal treatment after the first FAc-implant. Among the 35 eyes (47.9%) that did require additional intravitreal treatments, 33 (94.3%) of them received DEX-implants. For the latter, the mean time between FAc-implant and the first subsequent DEX-implant was 5.8 (1.7) months. In the total cohort, the mean number of DEX-implant per year per patient decreased from 3.1 (0.8) before FAc-implant to 0.8 (0.2) after FAc-implant (*p* < 0.001). At 12 months, the probability of not receiving a new rescue injection of DEX-implant was 54.0% [43.0%;67.0%] (Fig. 2).

Finally, 6 eyes (8.2%) had a repeated 0.19-mg FAc-implant, at a mean of 25.7 (3.0) months after the first implant: in 4 of these eyes, the subsequent FAc-implant was injected due to recurrent MO (at least 21 months after the first FAc-implant) while in 2 eyes the injection was performed prophylactically, in anticipation of a decrease in treatment effectiveness.

In terms of safety, all 73 eyes (100%) were pseudophakic prior to FAc-implant (Table 1); cataract development following FAc-implant could thus not be studied.

**Fig. 3:**
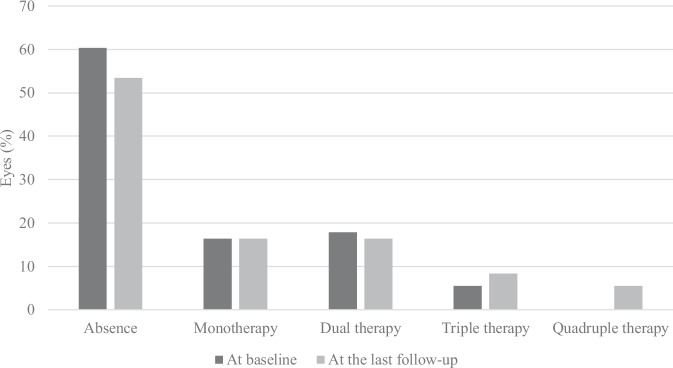
Distribution of eyes treated and untreated with IOP-lowering eye drops at baseline and at the last follow-up. IOP intraocular pressure.

At baseline, the mean IOP was 11.4 (4.2) mmHg, and reached a maximum at 1 month post FAcimplant. Although the median difference in IOP was statistically significant from baseline up until 12 months, it remained statistically unchanged from 15 months to 24 months (Supplementary Table 1). At last follow-up, 39 eyes (53.4%) were not treated by IOP-lowering eye drops (Fig. 3). An IOP-lowering treatment was introduced for 7 eyes (9.6%) while for 11 eyes (15.1%) already treated, the number of IOP-lowering eye drops was increased. The most commonly used IOP-lowering eye drops were beta-blockers, carbonic anhydrase inhibitors, and prostaglandins. More rarely, when the IOP was particularly high, alpha-2 adrenergic agonists and carbonic anhydrase inhibitors were used orally for 7 (9.6%) and 3 eyes (4.1%), respectively. Selective laser trabeculoplasty was performed in 7 eyes (9.6%) at a mean of 10.0 (3.2) months after FAc-implant. Two eyes (2.7%) underwent IOP-lowering surgery during follow-up, 3 and 10 months after FAc-implant, despite treatment with IOP-lowering medical therapy. Both had known glaucoma and were treated with IOP-lowering drops (2 and 3, respectively) before FAc-implant. Two eyes (2.7%) had a resolved hypotony during followup with Seidel signs, managed by medical treatment (one already had repeated hypotony under DEX-implant). Throughout follow-up, 23 eyes (31.5%) had increased IOP ≥ 21 mmHg. Eight eyes (11.0%) had OHT (IOP ≥ 25 mmHg or an increase in IOP ≥ 10 mmHg): 4 at the first month after FAc-implant and 4 after the ninth. All had already been treated with at least one IOPlowering eye drop prior to FAc-implant. A significant association was found between eyes presenting with increased IOP ≥ 21 mmHg before FAc-implant (after DEX-implant) and those with increased IOP ≥ 21 mmHg after FAc-implant (*p* = 0.002).

A significant decrease in median RNFL between baseline and follow-up visits was observed (Supplementary Table 1). The median difference in RNFL was -6.0 [-10.0;-1.3] μm at 6 months (*p* < 0.001), -7.0 [-13.0;0.0] μm at 12 months (*p* < 0.001), -8.0 [-18.3;-2.3] μm at 18 months (*p* = 0.001), and -10.0 [-15.5;-4.0] μm at 24 months (*p* = 0.022). The number of eyes affected by confirmed glaucoma increased from 20.3% before FAc-implant to 27.1% at last follow-up.

Finally, one FAc-implant was removed during vitrectomy surgery due to lens implant dislocation requiring explantation and then reimplantation of an iris fixation implant, contraindicating the use of intravitreal devices. Four other patients underwent vitrectomy surgery after FAc-implant (3 for epiretinal membrane peeling and one for management of intravitreal haemorrhage after injection of a rescue DEX-implant), without removing the FAcimplant. In the total cohort, no anterior chamber migration of the FAc-implant was observed and no endophthalmitis or vasculitis occurred during follow-up. One case of FAc-implant embedded in the posterior capsule directly after implantation was observed and released by neodymium-doped yttrium aluminium garnet laser.

## Discussion

The present real-life study confirms the results of previous randomised controlled studies [7, 8] demonstrating the effectiveness and safety of FAc-implant in chronic inflammatory MO. The present study contributes rare comparative data by evaluating uveitic and postoperative MO in the same cohort, due to their potential pathophysiological and therapeutic similarities.

In terms of functional outcomes, the present study showed an improvement in BCVA at each follow-up visit, with almost three-quarters of patients achieving a gain ≥5 letters. This improvement was maintained at ≥69 letters throughout the follow-up period, in line with the findings of Pavesio et al. who demonstrated a mean BCVA above 70 letters during a 3-year follow-up in a cohort of patients with uveitis [12]. Regarding anatomical outcomes, CMT was also significantly reduced throughout the follow-up period, very shortly after FAc-implant, similarly to previous findings [13–16]. This rapid kinetic, from the start, can be explained by the action of the DEX-implant, as the two molecules herein were administered almost simultaneously in the majority of patients. Interestingly, the decrease in CMT was sustained during the follow-up with a reduction in thickness fluctuation; this is of importance as CMT fluctuations are known to be associated with poorer visual outcomes in eyes with MO [9]. Furthermore, it is worth noting that the effect of the FAc-implant in terms of anatomical and functional outcomes did not differ significantly between uveitic and postoperative MO groups. Although the FAc-implant has not yet been approved by the health authorities for the treatment of postoperative MO, its use in this context is supported by its pathophysiology and the good results obtained in some series [15, 17–20]. For example, Chronopoulos et al. found a significant improvement in CMT and BCVA up to 2 years post-FAc-implant in a cohort of 16 patients with postoperative MO [18]. The effectiveness of the FAc-implant in this postoperative indication is explained by the proinflammatory cytokines found in the vitreous of patients with postoperative MO [4]. Since the DEX-implant demonstrated its effectiveness and safety at 12 months in the treatment of postoperative MO (EPISODIC-2 study) [21], and given the likely inflammatory contribution to postoperative MO, the use of corticosteroids, including FAc-implant, could constitute an interesting therapeutic strategy in this setting. Based on these findings, FAc-implant should not be considered as a bolus treatment but rather as a basal background therapy that helps stabilise the disease and limit the fluctuations, preferably in patients sensitive to prior corticosteroid therapy [10, 11]. The simultaneous administration (within the same month) of DEX-implant and FAc-implant should thus enable to rapidly treat the MO recurrence while waiting for the pharmacological effectiveness of the FAc-implant that will ensure long-term treatment. This proposed treatment paradigm, used in the majority of the present cohort, follows the recent consensus guidelines on the use of FAc-implant [10, 11]. However, the concomitant use of DEXimplant, in combination with FAc-implant may bias the interpretation of the results regarding the effectiveness of the FAc-implant during early follow-up. This may also be the case for other concomitant or adjuvant therapies, including DEX-implants, systemic treatment, and periocular steroids since such treatments can lead to transient or sustained reductions in CMT and improvements in BCVA, independently of the FAc-implant. More specifically, DEX-implants may provide rapid oedema resolution in uveitic eyes, potentially enhancing early CMT reduction and visual gain [22, 23], while systemic or periocular corticosteroids can contribute to oedema improvement. All additional treatments were carefully documented, including their timing relative to FAc-implant. Although administered when clinically indicated, these concomitant therapies should be considered when interpreting functional and anatomical outcomes. Importantly, the sustained effect observed in the present cohort, beyond the typical effect duration of adjunctive therapies, supports the long-term effectiveness of the FAc-implant in both uveitic and postoperative inflammatory MO.

Regarding additional treatment following FAc-implant, almost half of the patients herein did not need additional treatment over the follow-up period. More precisely, the survival without DEX-implant at 12-month follow-up was 54%, a finding less conclusive than those of Jabbour et al. [24] and Motloch et al. [15] who reported a rescue DEX-implant in the first 12 months in 23% of uveitic eyes and 28.6% of postoperative MO eyes, respectively.

Regarding safety, the FAc-implant was generally well tolerated during follow-up, with safety data in the same proportions as in the literature cohorts [7, 15, 24, 25]. Although a change in median IOP compared to baseline was observed during the first 15 months, no peak was observed and the values remained in the normal range. It is well known that steroids can induce OHT and RNFL thinning reflecting secondary glaucoma [26]; OHT was observed in 8 eyes herein, at about a mean of 7 months after FAc-implant. This observation is in line with both the IRISS study [27] and the work of Mathis et al. [25]. However, it should be noted that 4 of the eyes that developed OHT herein showed early OHT, one month after FAc-implant. In half of these cases, the FAcimplant had been administered concomitantly to the DEX-implant; given the kinetics of onset, it is likely that the DEX-implant led to the OHT in these eyes. Nevertheless, the other 2 eyes with OHT had not received a DEX-implant within the 4 months preceding the episode. It is also important to note that OHT occurred exclusively in eyes already treated with IOP-lowering eye drops before FAc-implant. Moreover, the findings herein suggest a possible predictability of increased IOP ≥ 21 mmHg after FAc-implant according to increased IOP occurrence after DEXimplant. This is reinforced by the results of the Medisoft^**®**^ and USER studies showing that IOP response to prior DEX-implant had a very good positive predictive value (around 90%) for IOP response to FAc-implant [28, 29]. A careful selection of patients according to the presence of hypotensive treatment at baseline and previous OHT and even IOP ≥ 21 mmHg, under DEXimplant thus seems essential. In both uveitic and postoperative MO, a decline in RNFL, which is correlated with IOP variations, was observed herein. As could be expected, this decline was more pronounced in the uveitic group. Uveitis is indeed recognised to be a significant confounding factor in the assessment of RNFL thickness, as active inflammation can cause swelling of the optic nerve head, resulting in artificially increased RNFL values. Moore et al. [30] reported that, in 19 eyes with active non-glaucomatous uveitis, the mean overall and sectoral RNFL measurements exceeded the 95th percentile of the normal range. Furthermore, in glaucomatous eyes with active or quiescent uveitis, the mean overall RNFL thickness was greater than that reported in eyes with equivalent stages of non-uveitic glaucoma [30]. Therefore, once inflammation is adequately controlled, RNFL and other SD-OCT measurements may decrease, as observed in the present cohort. In contrast, we noted a slight increase in RNFL thickness in eyes with established glaucoma at the end of follow-up, whereas values remained stable in the Moll-Udina cohort [31]. In such situations, or when uncertainty persists, visual field testing may be useful. Two patients required filtering surgery for IOP uncontrolled by medical treatment and 2 had post-implant hypotony. These serious side effects may seem relatively frequent but have been reported in similar proportions in several real-life studies [32–34] and in randomised clinical trials [7, 8]. Overall, the effectiveness of FAc-implant should be evaluated by discussing the risk/benefit balance with the patient, by taking into account the reduction in treatment burden, the risk of OHT, and the need for regular ophthalmic evaluation.

The present study has several limitations. First, regarding the study design, its retrospective nature limited the exhaustiveness of the data collection and analysis, although few missing data were reported. The absence of a control group and the lack of masking are therefore a limiting factor inherent to the design. Furthermore, the observational design implies heterogeneity in follow-up and imaging intervals, as well as in the criteria used to propose additional therapies. Thus, the gradual decrease in the number of patients analysed over time could introduce a potential time-effect bias and an attrition bias, since the entire study population did not have the same follow-up time and thus unequal visit completion. With regard to treatment, there is also a potential temporal bias introduced by the concomitant treatment with DEX-implant as well as confounding factors due to systemic therapies received by the patient. Secondly, it would have been interesting to investigate the SD-OCT anatomical prognostic factors of MO recurrence; indeed, it has been shown that disorganisation of the inner retinal layers and hyperreflective foci were associated with a higher likelihood of requiring a rescue DEXimplant [24]. It would thus be interesting to investigate this point further as the early detection of MO recurrence, through complementary examinations, would enable to administer a rescue DEX-implant earlier, before MO alters retinal cells and visual acuity is impacted. Finally, although the mean follow-up was about 17 months, the minimum 6-month follow-up period can be viewed as relatively short to evaluate FAc-implant effectiveness, since its theoretical duration of action is around 36 months; the 24-month follow-up data should be interpreted with caution given the small number of patients at that time point.

In conclusion, the present findings show the lasting anatomical and functional benefit of the FAc-implant in patients with chronic inflammatory MO, with a reduction in the therapeutic burden of repeated intravitreal injections. This treatment strategy could thus be considered in carefully selected patients who have responded well and safely to prior DEX-implant, with the aim to limit additional treatments and visits, prevent the recurrence of inflammatory MO, and reduce anatomical fluctuations, which are crucial for the patient’s visual prognosis. Safety data were similar to those in the literature.

## Summary

### What was known before

- Efficacy of the fluocinolone acetonide implant in the treatment of diabetic and uveitic macular oedema (clinical trials).
- Few real-world studies regarding postoperative macular oedema with small patient cohorts.
- A reassuring safety profile of the fluocinolone acetonide implant in diabetic and uveitic indications.

### What this study adds

- The study:
- supports the use of the FAc-implant in postoperative macular oedema even though it does not yet have marketing authorisation.
- compares the efficacy criteria between the two inflammatory origins (uveitic and postoperative): which has not previously been carried out.
- presents the results using innovative figures.
- represents one of the largest real-world cohorts reported in the literature in this field.
- focuses on the quantification of the therapeutic burden, which is significantly reduced for the patient.

## Supplementary information

Supplementary Figure 1

Supplementary Table 1

Supplementary Table 2

## Data Availability

The datasets generated during and/or analysed during the current study are available from the corresponding author on reasonable request.
