# Supplementary material for: Use of intravitreal fluocinolone acetonide implant in inflammatory macular oedema
Source: Eye (Lond). 2026 Mar 19;40(8):1145–54. doi: 10.1038/s41433-026-04381-9 (PMC13195103; doi:10.1038/s41433-026-04381-9)
Supplement: Supplementary file 1 — Supplementary Figure 1 [file 41433_2026_4381_MOESM1_ESM.pdf]

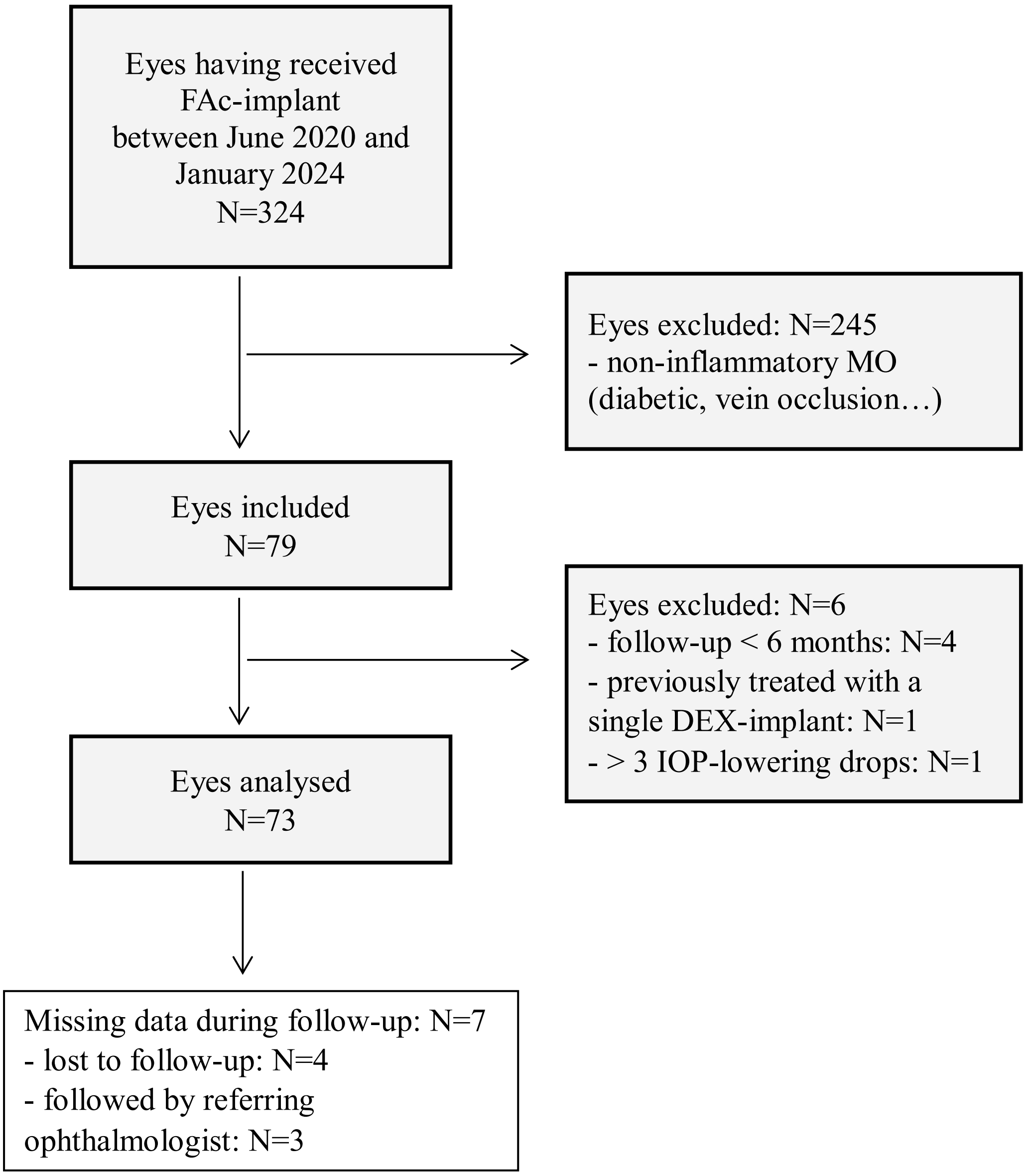

<u>Supplementary Figure 1: Study flow chart.</u>

DEX-implant= dexamethasone implant, FAc-implant= fluocinolone acetonide implant, IOP= intraocular pressure, MO= macular oedema, N=number.
