# Supplementary material for: Use of intravitreal fluocinolone acetonide implant in inflammatory macular oedema
Source: Eye (Lond). 2026 Mar 19;40(8):1145–54. doi: 10.1038/s41433-026-04381-9 (PMC13195103; doi:10.1038/s41433-026-04381-9)
Supplement: Supplementary file 2 — Supplementary Table 1 [file 41433_2026_4381_MOESM2_ESM.docx]

Supplementary Table 1: Best-corrected visual acuity, central macular thickness, intraocular pressure, and retinal nerve fibre layer during follow-up in the total cohort.

|  | **M0**  **N=73** | **M1**  **N=60** | **M3**  **N=70** | **M6**  **N=71** | **M9**  **N=63** | **M12**  **N=53** | **M15**  **N=45** | **M18**  **N=33** | **M21**  **N=22** | **M24**  **N=13** |
| --- | --- | --- | --- | --- | --- | --- | --- | --- | --- | --- |
| **Mean BCVA, letters (SD)** | 65.3  (15.3) | 71.4  (14.0) | 69.5  (14.2) | 69.6  (15.0) | 68.5  (15.3) | 70.5  (14.9) | 69.9  (13.0) | 72.5  (10.3) | 78.2  (6.5) | 73.5  (8.4) |
| **Median BCVA, letters [IQR]** | 69.5  [60.0;78.0] | 77.0  [65.0;82.0] | 74.0  [65.0;80.0] | 75.0  [62.0;80.0] | 74.0  [60.0;79.0] | 75.0  [65.0;80.0] | 75.0  [65.0;78.0] | 75.0  [68.0;79.0] | 80.0  [75.0;84.0] | 75.0  [67.0;79.0] |
| **Median difference in BCVA,**  **letters [IQR]** | 0  [0.0;0.0] | 5.0  [0.0;10.0]  **p=0.003** | 3.0  [0.0;8.3]  **p<0.001** | 4.0  [0.0;10.0]  **p<0.001** | 1.0  [0.0;8.0]  **p=0.002** | 2.0  [0.0;9.0]  **p=0.006** | 2.0  [-1.0;10.0]  **p=0.013** | 5.0  [0.0;10.0]  **p=0.010** | 6.5  [0.5;10.8]  **p=0.013** | 5.0  [0.0;9.0]  **p=0.043** |
| **Mean CMT,**  µ**m (SD)** | 425.4  (110.7) | 295.1  (59.3) | 315.2  (69.9) | 317.7  (74.8) | 315.5  (72.3) | 312.4  (77.5) | 320.2  (77.7) | 308.2  (61.8) | 298.7  (54.1) | 286.2  (42.7) |
| **Median CMT,**  µ**m [IQR]** | 409.0  [341.0;  488.0] | 297.0  [262.0;  321.0] | 306.0  [269.0;  345.0] | 305.0  [271.0;  341.0] | 303.0  [265.0;  358.0] | 302.0  [268.0;  342.0] | 300.0  [269.0;  344.0] | 302.0  [268.0;  344.0] | 301.0  [259.0;  341.0] | 283.0  [259.0;  300.0] |
| **Median difference in CMT,**  µ**m [IQR]** | 0  [0.0;0.0] | -74.5  [-193.8;  -32.0]  **p<0.001** | -70.0  [-181.5;  27.5]  **p<0.001** | -71.0  [-181.3;  -25.5]  **p<0.001** | -71.0  [-177.3;  -36.5]  **p<0.001** | -71.0  [-195.0;  -30.5]  **p<0.001** | -90.0  [-196.5;  -40.0]  **p<0.001** | -75.0  [-194.0;  -43.0]  **p<0.001** | -88.5  [-212.5;  -58.0]  **p<0.001** | -154.0  [-253.0;  -110.0]  **p<0.001** |
| **Mean IOP,**  **mmHg (SD)** | 11.4  (4.2) | 14.7  (6.4) | 13.3  (4.9) | 13.9  (5.0) | 13.6  (4.9) | 12.8  (4.5) | 12.7  (5.1) | 13.2  (5.1) | 12.1  (3.7) | 11.8  (3.2) |
| **Median IOP, mmHg [IQR]** | 11.0  [9.0;13.0] | 13.0  [10.0;18.0] | 12.0  [10.0;16.0] | 13.0  [11.0;17.0] | 12.0  [10.0;17.0] | 11.0  [10.0;15.0] | 12.0  [9.0;16.0] | 13.0  [10.0;16.0] | 12.0  [10.0;14.0] | 13.0  [9.0;14.0] |
| **Median difference in IOP,**  **mmHg [IQR]** | 0  [0.0;0.0] | 3.0  [-0.6;7.0]  **p<0.001** | 1.0  [-1.0;4.4]  **p<0.001** | 2.0  [0.0;4.0]  **p<0.001** | 1.2  [-1.0;4.0]  **p=0.002** | 1.0  [-1.0;4.0]  **p=0.03** | 2.0  [-2.0;6.5]  **p=0.08** | 1.0  [-1.0;6.0]  **p=0.07** | 1.0  [-1.0;4.0]  **p=0.40** | -1.0  [-3.0;4.0]  **p>0.90** |
| **Mean RNFL,**  **μm (SD)** | 104.2  (24.7) | \| 102.8  (20.1) \| \| --- \| | 101.1  (24.5) | 98.0  (25.1) | 100.7  (20.3) | 99.1  (22.7) | 97.7  (20.4) | 99.2  (27.3) | 99.8  (31.2) | 100.8  (29.9) |
| **Median RNFL, μm [IQR]** | 104.0  [90.0;120.0] | 106.0  [89.0;115.5] | 104.0  [87.0;112.0] | 99.0  [81.3;113.8] | 102.5  [86.0;114.8] | 100.0  [82.5;109.5] | 96.0  [82.5;109.5] | 98.0  [77.5;106.5] | 97.0  [77.5;102.3] | 92.0  [82.0;115.0] |
| **Median difference in RNFL,**  **μm [IQR]** | 0  [0.0;0.0] | -4.0  [-8.0;-0.5]  **p<0.001** | -3.0  [-8.0;0.0]  **p<0.001** | -5.5  [-10.0;-1.3]  **p<0.001** | -3.0  [-11.5;0.0]  **p=0.002** | -7.0  [-13.0;0.0]  **p<0.001** | -6.0  [-13.0;0.0]  **p<0.001** | -7.5  [-18.3;-2.3]  **p=0.001** | -8.0  [-15.0;-3.0]  **p<0.001** | -9.5  [-15.5;-4.0]  **p=0.022** |

The columns highlighted in grey correspond to the months of follow-up visits post-FAc-implant retained for the primary outcome analysis.

P-values were calculated using Wilcoxon signed rank test and corrected following Holm’s method to control for the family-wise error rate.

BCVA= best-corrected visual acuity, CMT= central macular thickness, IOP= intraocular pressure, IQR= interquartile range, M= month of follow-up visit, N= number of eyes analysed, RNFL= retinal nerve fiber layer, SD= standard deviation.
