# Supplementary material for: Use of intravitreal fluocinolone acetonide implant in inflammatory macular oedema
Source: Eye (Lond). 2026 Mar 19;40(8):1145–54. doi: 10.1038/s41433-026-04381-9 (PMC13195103; doi:10.1038/s41433-026-04381-9)
Supplement: Supplementary file 3 — Supplementary Table 2 [file 41433_2026_4381_MOESM3_ESM.docx]

Supplementary Table 2: Estimated coefficients for the GAMLSS model.

1. For the total cohort (cf Figure 2).

| Parameter | Term | Coefficient | 95% CI | P-value |
| --- | --- | --- | --- | --- |
| CMT | Mean outcome before FAc-implant | 351.43 | [341.22;361.63] | <0.001 |
| CMT | Trend of outcome per year before FAc-implant | 2.13 | [-12.89;17.14] | 0.8 |
| CMT | Mean change in outcome immediately after FAc-implant | -10.35 | [-20.85;0.14] | 0.054 |
| CMT | Change in trend of outcome per year after FAc-implant | -16.08 | [-31.32;-0.85] | 0.039 |
| CMT | Within patient SD of outcome before FAc-implant | 4.21 | [4.12;4.29] | <0.001 |
| **CMT** | **Change in within patient SD after FAc-implant (vs. before)** | **-0.62** | **[-0.73;-0.52]** | **<0.001** |
| BCVA | Mean outcome before FAc-implant | 68.59 | [66.87;70.32] | <0.001 |
| BCVA | Trend of outcome per year before FAc-implant | 0.55 | [-1.98;3.08] | 0.7 |
| BCVA | Mean change in outcome immediately after FAc-implant | 0.82 | [-0.96;2.60] | 0.4 |
| BCVA | Change in trend of outcome per year after FAc-implant | 0.38 | [-2.19;2.95] | 0.8 |
| BCVA | Within patient SD of outcome before FAc-implant | 1.98 | [1.89;2.06] | <0.001 |
| **BCVA** | **Change in within patient SD after FAc-implant (vs. before)** | **-0.60** | **[-0.71;-0.50]** | **<0.001** |

Interest coefficients are highlighted in bold.

1. With stratification by aetiology of MO, other associated cause of MO, tractional epiretinal membrane.

|  |  |  | Model results for $\mu_{i}$ / Mean model | | | Model results for $\sigma_{i}$ / Variance model | | |
| --- | --- | --- | --- | --- | --- | --- | --- | --- |
| Stratification | Outcome | Coefficient | Value | 95% CI | P-value | Value | 95% CI | P-value |
| Aetiology of MO   - Uveitic (ref) - Postoperative | CMT | $\beta_{0}$ | 336.7 | [325.9;347.5] | <0.001 | 3.9 | [3.8;4.1] | <0.001 |
|  |  | $\beta_{1}$ | 5.2 | [-10.3;20.7] | 0.5 |  |  |  |
|  |  | $\beta_{2}$ | -13.2 | [-24.4;-2.0] | 0.021 | -0.7 | [-0.8;-0.5] | <0.001 |
|  |  | $\beta_{3}$ | 28.2 | [17.4;39.0] | <0.001 | 0.5 | [0.3;0.7] | <0.001 |
|  |  | $\beta_{4}$ | -17.9 | [-33.5;-2.3] | 0.025 |  |  |  |
|  |  | $\boldsymbol{\beta}_{\boldsymbol{5}}$ | **8.7** | **[-5.1;22.5]** | **0.2** | **0.1** | **[-0.1;0.3]** | **0.5** |
|  |  | $\beta_{6}$ | -9.0 | [-15.9;-2.1] | 0.011 |  |  |  |
|  | BCVA | $\beta_{0}$ | 69.0 | [67.0;70.9] | <0.001 | 2.0 | [1.9;2.2] | <0.001 |
|  |  | $\beta_{1}$ | 0.5 | [-2.0;3.1] | 0.7 |  |  |  |
|  |  | $\beta_{2}$ | 0.1 | [-2.0;2.3] | >0.9 | -0.6 | [-0.8;-0.5] | <0.001 |
|  |  | $\beta_{3}$ | -0.4 | [-2.0;1.1] | 0.6 | -0.1 | [-0.3;0.1] | 0.2 |
|  |  | $\beta_{4}$ | 0.3 | [-2.2;2.8] | 0.8 |  |  |  |
|  |  | $\boldsymbol{\beta}_{\boldsymbol{5}}$ | **1.0** | **[-1.0;3.0]** | **0.3** | **0.1** | **[-0.2;0.3]** | **0.6** |
|  |  | $\beta_{6}$ | 0.2 | [-0.7;1.1] | 0.6 |  |  |  |
| Other associated cause of MO  (0/1) | CMT | $\beta_{0}$ | 353.7 | [343.6;363.9] | <0.001 | 4.2 | [4.2;4.3] | <0.001 |
|  |  | $\beta_{1}$ | 2.9 | [-11.9;17.7] | 0.7 |  |  |  |
|  |  | $\beta_{2}$ | -9.7 | [-20.2;0.8] | 0.070 | -0.6 | [-0.7;-0.5] | <0.001 |
|  |  | $\beta_{3}$ | -22.3 | [-35.0;-9.5] | <0.001 | -0.4 | [-0.7;-0.1] | 0.003 |
|  |  | $\beta_{4}$ | -15.9 | [-30.9;-0.9] | 0.037 |  |  |  |
|  |  | $\boldsymbol{\beta}_{\boldsymbol{5}}$ | **-5.0** | **[-20.7;10.8]** | **0.5** | **-0.1** | **[-0.4;0.2]** | **0.6** |
|  |  | $\beta_{6}$ | -5.3 | [-11.9;1.4] | 0.12 |  |  |  |
|  | BCVA | $\beta_{0}$ | 69.1 | [67.3;70.8] | <0.001 | 2.0 | [1.9;2.1] | <0.001 |
|  |  | $\beta_{1}$ | 0.5 | [-2.0;3.0] | 0.7 |  |  |  |
|  |  | $\beta_{2}$ | 0.9 | [-0.9;2.7] | 0.3 | -0.6 | [-0.8;-0.5] | <0.001 |
|  |  | $\beta_{3}$ | -6.5 | [-8.9;-4.0] | <0.001 | -0.2 | [-0.5;0.1] | 0.13 |
|  |  | $\beta_{4}$ | 0.5 | [-2.0;3.1] | 0.7 |  |  |  |
|  |  | $\boldsymbol{\beta}_{\boldsymbol{5}}$ | **0.9** | **[-2.5;4.4]** | **0.6** | **0.4** | **[0.0;0.7]** | **0.049** |
|  |  | $\beta_{6}$ | -2.1 | [-3.8;-0.4] | 0.015 |  |  |  |
| Tractional epiretinal membrane  (0/1) | CMT | $\beta_{0}$ | 341.7 | [331.2;352.2] | <0.001 | 4.3 | [4.2;4.4] | <0.001 |
|  |  | $\beta_{1}$ | -0.7 | [-15.1;13.7] | >0.9 |  |  |  |
|  |  | $\beta_{2}$ | -9.9 | [-21.1;1.2] | 0.081 | -0.7 | [-0.8;-0.5] | <0.001 |
|  |  | $\beta_{3}$ | 56.9 | [48.2;65.7] | <0.001 | -0.3 | [-0.5;-0.1] | 0.005 |
|  |  | $\beta_{4}$ | -14.8 | [-29.2;-0.3] | 0.045 |  |  |  |
|  |  | $\boldsymbol{\beta}_{\boldsymbol{5}}$ | **-1.0** | **[-12.5;10.5]** | **0.9** | **0.2** | **[0.0;0.5]** | **0.079** |
|  |  | $\beta_{6}$ | 4.0 | [-1.7;9.6] | 0.2 |  |  |  |
|  | BCVA | $\beta_{0}$ | 69.1 | [67.4;70.8] | <0.001 | 1.9 | [1.8;2.0] | <0.001 |
|  |  | $\beta_{1}$ | 0.7 | [-1.8;3.2] | 0.6 |  |  |  |
|  |  | $\beta_{2}$ | 0.9 | [-0.8;2.7] | 0.3 | -0.6 | [-0.7;-0.4] | <0.001 |
|  |  | $\beta_{3}$ | -2.7 | [-5.2;-0.2] | 0.036 | 0.3 | [0.1;0.5] | 0.008 |
|  |  | $\beta_{4}$ | 0.3 | [-2.2;2.8] | 0.8 |  |  |  |
|  |  | $\boldsymbol{\beta}_{\boldsymbol{5}}$ | **-0.9** | **[-4.1;2.3]** | **0.6** | **-0.2** | **[-0.5;0.1]** | **0.2** |
|  |  | $\beta_{6}$ | -1.3 | [-2.9;0.3] | 0.12 |  |  |  |

The coefficients associated with treatment effect (FAc-implant) modification from stratification variables are stressed in bold

**Stratification**: which variable stratifying the analysis was added here (aetiology of MO, other associated cause of MO, tractional epiretinal membrane)? In these stratified models, we seek to see whether the values estimated for the entire cohort change between subgroups.

o For aetiology: the results will be expressed in postoperative (analysed) compared to uveitic MO (reference).

o For other associated cause of MO: in yes (analysed) compared to no (reference).

o For tractional epiretinal membrane: same.

**Outcome:** CMT and BCVA.

**Term:** indicates which variable the results (estimate, 95% CI and p-value) relate to and how this result should be interpreted.

$\beta_{0}$ (Intercept): value in an eye just before FAc-implant, whose stratification variable is on its reference modality (uveitic MO/no/no, as applicable).

$\beta_{1}$ delay0: rate of change in the outcome over time before FAc-implant in the reference modality.

$\beta_{2}$ n_implant1: effect of FAc-implant in the reference modality.

$\beta_{3}$ stratification (aetiology of MO, other cause of MO, tractional epiretinal membrane): difference in outcome in the analysed modality of the stratification variable compared to the reference.

$\beta_{4}$ delay0:n_implant1: change in slope after FAc-implant compared to before.

$\beta_{5}$n_implant1:stratification: change in the effect of FAc-implant in the analysed stratification modality compared to the reference group (n_implant1+n_implant1:stratification gives the effect of the implant in the analysed modality).

$\beta_{6}$ delay0:stratification: rate of change in the outcome after FAc-implant in the analysed stratification modality compared to the reference group

Two sets of results are provided: one for mean model and a second for variance model (intra-eye fluctuation).

The p-value tests whether the stratification variables modify the effect of FAc-implant, either on the outcome measure directly (mean model) or on the variability of the outcome (variance model).

o A negative p-value (p>0.05) confirms the presence of a homogeneous effect of FAc-implant

o A positive p-value (p<0.05) suggests a lesser or greater benefit of FAc-implant in one subgroup than another.

BCVA= best-corrected visual acuity, CI=confidence interval, CMT=central macular thickness, FAc-implant= fluocinolone acetonide implant, GAMLSS= generalised additive model for location, scale, and shape, MO=macular oedema, SD= standard deviation.
